# Who owns your data? Part III challenges to academic freedom

**DOI:** 10.1002/acm2.12443

**Published:** 2018-08-13

**Authors:** Michael D. Mills

**Affiliations:** ^1^ Radiation Oncology University of Louisville Louisville KY USA

What claim does your employer have on your publications? On your data? On your intellectual properties? If you teach, research, or publish something controversial, just how much freedom do you have to do what you wish? If you want to leave your institution for new employment, what are you allowed to take with you?

I considered a number of definitions of academic freedom, and I like this succinct one from Wikipedia the best: “Academic freedom is the conviction that the freedom of inquiry by faculty members is essential to the mission of the academy as well as the principles of academia, and that scholars should have freedom to teach or communicate ideas or facts (including those that are inconvenient to external political groups or to authorities) without being targeted for repression, job loss, or imprisonment.” https://en.wikipedia.org/wiki/Academic_freedom. Really, this is a good idea not just for universities. A diversity of ideas makes for better businesses and other organizations as well.

Academic freedom exists first, for the good of the people, then to support academic excellence through diversity, and finally to protect the employment, security, and quality of life interests of the scholars. Academic freedom will mean something different altogether, depending on the individual and the venue. For some, it means the right to say what they wish in a classroom, regardless of the context, but there are limitations to this. As an example, teaching the doctrines of phrenology might be acceptable in a class on historical medicine, but not in a contemporary class on neuroanatomy. Penn State professors made a heroic attempt to further cement the principles for academic freedom in modern academia back in 2010. https://www.insidehighered.com/views/2010/12/21/defining-academic-freedom.

Here are a few of its main points: Academic freedom means that both faculty members and students can engage in intellectual debate without fear of censorship or retaliation. Academic freedom in teaching means that both faculty members and students can make comparisons and contrasts between subjects taught in a course and any field of human knowledge or period of history. Academic freedom means that the political, religious, or philosophical beliefs of politicians, administrators, and members of the public cannot be imposed on students or faculty.

What does academic freedom mean in the context of open access publishing, and what are its implications for ownership of data? Here, we are in the midst of debate and controversy. In 2008, Harvard University faculty voted unanimously “to make available his or her scholarly articles and to exercise the copyright in those articles. In legal terms, the permission granted by each faculty member is a nonexclusive, irrevocable, paid‐up, worldwide license to exercise any and all rights under copyright relating to each of his or her scholarly articles, in any medium, and to authorize others to do the same, provided that the articles are not sold for a profit.” https://osc.hul.harvard.edu/policies/fas/ Many scholarly communities have followed suit.

However, not all agree that universities have a right to such secondary publication of faculty articles. Seventeen academics at the University of Konstanz have objected and taken the open access policy to the German courts. https://www.uni-konstanz.de/en/university/news-and-media/current-announcements/news/news-in-detail/open-access-satzung-auf-juristischem-pruefstand/. Not everyone agrees that articles that do not need to be open access by law should nevertheless be published open access regardless of the desires of the author. Authors may have justifiable reasons to prefer publishing in a venue that does not have unrestricted access, such as the inclusion of intellectual property, controversial data, or political perspectives that are sensitive. Also, the universities’ right to publish all faculty articles open access implies some kind and degree of ownership over the article. This is true even if the copyright is assigned to the authors. Should academics, therefore, be forced to publish open access by university mandate? Who really controls the copyright?

In a larger context, the move toward universal open access publication of research papers continues relentlessly. On June 21, 2017, a US court granted Elsevier a $15 million‐dollar judgment against Sci‐Hub for copyright infringement. However, Sci‐Hub is based in Russia and the founder, Alexandra Elbakyan, apparently does not have any assets in the US. https://www.nature.com/news/us-court-grants-elsevier-millions-in-damages-from-sci-hub-1.22196. It seems unlikely that Elsevier will see any of these funds, despite that fact, the judgment is likely small compared to the loss of restricted access to their many journal articles and other publications over the years. Also, consider that despite this ruling, Sci‐Hub is very popular. Hence, will anything change? Not likely. What this means for the academics at the University of Konstanz is their apparent quest to avoid publication in open access journals is probably doomed in the long run.

Another area of contention is the ownership of the data from which scholarly work is derived, analyzed, interpreted, and eventually described in a publication. If a faculty member moves from one university to another institution, who controls the data and its future use? If the data are based on protected health information (PHI), there are strict legal limitations on how that data may be shared. In this event, usually the university owns the data, even if the data are anonymized. There may be exceptions to this general rule that involve specific university policy governing collaborations with scholars from outside institutions within the legal framework of HIPAA. These would need to be set up in advance of beginning projects involving such data. A scholar is responsible to understand what data may and may not be taken to new employment. If a scholar working with non‐PHI data has spent years building a repository with potentially valuable and significant intellectual property, there may be objections if and when the scholar leaves for other employment. In this case, the outcome would be negotiated on a case‐by‐case basis.

All of this is to say that academic freedom, however defined, is evolving and new perceptions and alliances are being formed and negotiated. As scholars, we need to be aware who owns our publications, our data, and our intellectual property. These policies are being hammered out at individual universities, nationally, and globally. It is important that we know these things before we consider new employment, where the local policies and enforcement may be an unwelcome surprise.

